# Exploring the cytotoxic potential of *Artemisia herba-alba* crude extract and key phytoconstituents using *in vitro* assays and molecular docking insights

**DOI:** 10.1371/journal.pone.0359068

**Published:** 2026-09-25

**Authors:** Lidia Kamal Al-Halaseh, Ali M. Al-Samydai, Said Moshawih, Moath Al-Qaraleh, Diana M. Al-Zregat, Zaid S. Amareen, Manal Nahar Al Soub, Nadine N. Abdelhadi, Samra Qaraghuli

**Affiliations:** 1 Department of Pharmaceutical Chemistry, Faculty of Pharmacy, Mutah University, Al-Karak, Jordan; 2 Pharmacological and Diagnostic Research Centre, Faculty of Pharmacy, Al-Ahliyya Amman University, Amman, Jordan; 3 Department of Medical Laboratory Sciences, Faculty of Science, Al-Balqa Applied University, Al-Salt, Jordan; 4 Department of Medicine, Faculty of Medicine, Mutah University, Al-Karak, Jordan; 5 Department of clinical pharmacy and pharmacy practice, Faculty of pharmacy, Aqaba University of Technology, Aqaba, Jordan; 6 Department of Pharmacognosy and Medicinal Plants, college of Pharmacy, Mustansiriya University, Baghdad, Iraq; Faculty of Medicine of Tunis, TUNISIA

## Abstract

The aim of the current research project is to evaluate the potential cytotoxicity of the aqueous extract of *Artemisia -albaherba* aerial parts against selected cancer cell lines. The widely grown plant material was collected and extracted. The crude aqueous extract was subjected to MTT analysis to assess potential cytotoxicity and cell viability. The cell lines utilized include: Normal Human Umbilical Vein Endothelial cell line EA (HUVEC; cat. no. CRL-1730; ATCC), Triple Negative Breast cancer cell line, MDA MB 231 (HTB-26), Human Pancreatic Ductal Adenocarcinoma cell line PANC1 (CRL-1469), Human Colorectal Carcinoma HCT 116 (CCL-247) cell lines. Cisplatin and doxorubicin serve as reference agents, and the media vehicle serves as a negative control. The extract was analysed by UHPLC/MS-MS. Three of the detected bioactive phytoconstituents, namely liquiritigenin, isofraxidin, and artemisinin, were docked against three cancer-associated protein targets corresponding to the three investigated cancer cell lines. The selected targets were TP53/p53 in the HCT-116 colorectal cancer cell line, AKT1 in the MDA cell line, and KRAS in the PANC-1 pancreatic cancer cell line. A marked decrease in cell viability was observed in the human colon and breast cancer cell lines at plant extract concentrations of 0.046–50 mg/mL. The highest cytotoxicity, around 45%, was observed against human colon cancer cells at concentrations of 12.5–50 mg/mL. And the cytotoxicity was around 40% against breast cancer cells at a concentration of 50 mg/mL. The plant remedy exhibited moderate cytotoxicity against pancreatic cancer cells, with an IC_50_ of approximately 20% at the highest tested concentration. The biocompatibility of the aqueous extract was predicted; hence, normal epithelial cells retained their viability at 1.5 mg/mL and showed a moderate-to-weak effect at higher concentrations. The herbal extract exhibits the highest IC_50_ values against EA and PANC1 of 220–240 µg/mL, whereas against MDA-MB-231 and HCT-116 cells, the IC_50_ values are significantly lower at 80–100 µg/mL. This indicates greater sensitivity of breast and colon cancer cell lines to the natural product. The simulation analyses demonstrate that isofraxidin and liquiritigenin have better binding affinity to the selected proteins than artemisinin. The best overall complexes were TP53-isofraxidin and TP53-liquiritigenin, with docking scores around −8 kcal/mol, followed by KRAS-liquiritigenin and AKT1-isofraxidin. To sum up, *A. herb alba* has a potential selective anticancer effect that may be attributed to the phenolic compounds constituents. These promising findings warrant further study to elucidate its mechanism of action, safety profile, and to predict the optimal dosage formulation.

## Introduction

The genus *Artemisia* is classified under the tribe Anthemideae, subfamily Asteroideae, and the family Asteraceae. The tribe encompasses more than 500 species, distributed across Asia, Europe, and North America [1]. The genus comprises two branches *Dracunculus* and *Artemisia*. The latter contains around 400 species, characterized by an aromatic nature and a bitter taste [2]. The morphology of the genus *Artemisia* is described as alternate leaves, small, racemose, paniculate, or capitate capitula, inflorescence, involucral bracts that scattered in a few rows, ranges between flat to hemispherical, has no scales, sometimes hirsute, floret all tubular, has no pappus, obovoid achenes [3].

The Phytoanalysis of the genus *Artemisia* reveals the presence of several biologically active secondary metabolites, which include mainly terpenoids, polyphenols, and sterols [4,5]. The biological activities of the plant extract were attributed to these secondary metabolites. For centuries, Several *Artemisia* plants have been used in treating gynecological disorders, hence its generic name *Artemisia* is modified from the Greek word ‘Artemis’, which means Diana, the Greek Goddess [6].

Several pharmacological studies were performed to investigate and justify the traditional recipes and the folk use of plants belonging to the genus *Artemisia* in treating minor and major health disorders. Extract of *A. vulgaris* showed pharmacological activities against hepatic disorders, intestinal worms, irritability, gastric ulcer, and indigestion [7]. The ether extract of *A. nilagirica* leaves has antimicrobial activity against several pathogens, including *Phytophthora capsici* and the fungus *Drechslera sorokiniana* [8,9]. Moreover, the anti-ulcer activity of the ethanolic extract of the *A. nilagirica* was demonestrated after obtaining promising results in ulcer-induced experimental rodents [10].

Moreover, several species of the genus *Artemisia* have been investigated for their anti-inflammatory and anti-asthmatic effects. The essential oil of *A. argyi* showed a positive effect in alleviating asthmatic symptoms by regulating 5-LOX-CysLTs and IDO-1-KYN pathways [11]. The extract of *A. gmelinii* exhibits anti-inflammatory activity by impeding the degranulation of mast cells and exerting a balance between the immune cells TH1/TH2 [12].

In earlier times, limited options for treating cancer included surgery, radiation, and chemotherapeutic agents. After thoroughly understanding cancer progression, additional treatment lines were added to the treatment regimen, including immunomodulatory, photodynamic, stem cell, gene therapy, and biological molecules [13,14]. Oxidative stress and biosynthesis of Reactive Oxygen Species (ROS) are among the triggers of many serious diseases, including cancer. ROS could alter the structure of nucleic acids and other biomacromolecules, thereby negatively affecting the regulation of several transcription factors [15].

Several medicinal plants and their bioactive compounds have free radical scavenging activity, therefore, possess a protective and/or therapeutic activity against cancer. Some of these showed anti-proliferative and pro-apoptotic properties after being tested *in vitro* and *in vivo*. For example, curcumin displayed cytotoxicity against brain, lung, pancreas, liver, and blood cancers [16]. The natural alkaloid berberine could alleviate cancer by modulating several signaling pathways, while the flavonoid quercetin attaches to cellular receptors and disrupt signaling cascades [14]. A number of restrictions limited the clinical implementing of these phyto-compounds in clinical applications including low-aqueous solubility and reduced bioavailability, hence, several attempts were performed to overcome these challenges by using nanoparticles, liposomes, and other advanced delivery systems [17,18].

Artemisia plants were explored as well for their cytotoxic and antiproliferative activities. In a murine study, the methanolic extract of *A. nilagirica* showed significant anticancer activity in comparison with vincristine [19]. Whereas, formulating *A. turcomanica* into nisomes showed improvement in the plant anticancer effect against MCF-7 cells, the apoptosis activity refers to modulating the expression of cancer-related genes [20]. Similarly, the expression of hepatic cancer-related genes TGFβ1 and MYC was controlled by *A. absinthium* extract [21]. A recent study revealed that a methanolic extract of *A. herba-alba* exhibited various degrees of cytotoxicity against several lines of colorectal cancerous cells through arresting the G2-M phase cell cycle, accompanied by a reduction in Cyclin B1 and CDK1 expression, in addition to inhibiting cancer progression pathways by modulating PI3K/AKT/mTOR [22].

The current study aims to underscore the selective cytotoxicity of the aqueous extract of *A. herba-alba*, cultivated from south Jordan, against several human cell lines: Pancreatic Ductal Adenocarcinoma (PANC1, CRL-1469), Triple Negative Breast Cancer (MDA MB 231, HTB-26), Colorectal Carcinoma (HCT 116, CCL-247), and Normal Human Umbilical Vein Endothelial (HUVEC, CRL-1730; ATCC). Moreover, the study aims to predict the preferred orientations and binding affinities of isofraxidin, liquiritigenin, and artemisinin to protein targets in each of the selected cancer cell lines.

## Materials and methods

### The plant material

The aerial parts of the plant were collected in the springtime from the Al-Aghwar region, south Jordan (131 Km south of Amman). The fresh plant was identified by taxonomists at the Royal Society for the Conservation of Nature (RSCN) in Amman, Jordan. The plant material was dried in shade, crushed, and macerated with continuous shaking at 25℃ for 3 successive days using a Mmemert shaker waterbath^®^. The filtered aq. The extract was lyophilized using a Benchtop Manifold Freeze Dryer from Millrock Technology®, UK. The plant identity was elucidated and assigned a reference number of RS/10/1/518. The preparation of the plant aqueous extract was performed in accordance with specifications in published reports [23,24].

### MTT-based cell viability and cytotoxicity assay

The cytotoxicity of the aqueous extract of *A. helba alba* against several cell lines was evaluated using the MTT (3-(4,5-dimethylthiazol-2-yl)-2,5-diphenyltetrazolium bromide assay). The examined cell lines are: Normal Human Umbilical Vein Endothelial cell line, EA (HUVEC; cat. no. CRL-1730; ATCC), Triple Negative Breast Cancer cell line, MDA MB 231 (HTB-26), Human Pancreatic Ductal Adenocarcinoma cell line, PANC1 (CRL-1469), and Human Colorectal Carcinoma cell line, HCT 116 (CCL-247). The cell lines were maintained in accordance with the supplier recommendations and routinely screened for mycoplasma to ensure research accuracy and reliability.

The cell density was optimized for each cell line to a range of 5–15 x 10^3^ cells/well in a volume of 100 µL media. To allow equilibrium, the cells were incubated for 24 h before starting the treatment. The protocol used was adopted from a previously published articles [25,26].

The seeded cells in 96-well flat-bottom plates were treated with geometric concentrations of the prepared lyophilized extract starting from a stock solution of 50 mg/mL water. After the incubation time, the MTT solution was added, and the mixture was incubated for 4 hours. Viable cells produced formazan crystals as metabolites. The resulting crystals were dissolved in DMSO. The IC values were determined by nonlinear regression using a four-parameter logistic (4PL) dose-response model. For cell lines in which the maximum observed inhibition did not exceed 50% across the tested dose range, the IC values presented reflect model estimates generated by curve fitting, not directly measured values. Background-corrected absorbance at λ_max_ = 590–630 nm values were used to determine the cells’ viability compared to vehicle control:


$$\begin{array}{c} Viability\left(\%\right)=\frac{A_{sa\text{mple}}-A_{bl\text{ank}}}{A_{v\text{ehicle}}-A_{bl\text{ank}}}\times 100\% \end{array}$$

The culture medium used to treat the cells served as the negative control in the experiment, normalizing cell viability and excluding any solvent-related effects. MTT assays were performed on the selected cell lines using two conventional anticancer drugs, namely: cisplatin and doxorubicin as reference chemotherapeutic agents. The assay was performed under identical conditions across the cell lines to those used in testing the different concentrations of the plant remedy.

To validate the experimental environment, a routine screening was performed to ensure the culture is free from mycoplasma contamination. Cell viability was assessed by confirming linearity between cell number and absorbance at 570 nm for each cell line before running the assay. Moreover, the assay conditions, including the cell density, incubation time, and reagent concentration, were optimized for each cell line. Three independent biological replicates and triplicate technical tests were performed for each cell line assay, and the average value ± standard deviation was reported.

### UHPLC/MS-MS analysis

The prepared aqueous extract was analysed using an Elute UHPLC coupled to a Bruker Impact II QTOMS. A Solo 2.0-C18 analytical column was used, and the mobile phase was water with formic acid (0.01%) and acetonitrile. The analysis was performed for 35 min in both negative and positive modes. The selected phytoconstituents: isofraxidin, liquiritigenin, and artemisinin were detected, and their peaks were extracted.

### Molecular docking analysis

#### Exploring the protein targets.

After screening the identified phytoconstituents in *A. herba-alba* from the literature, three active compounds were identified and then selected to perform molecular docking analysis: artemisinin, isofraxidin, and liquiritigenin. These compounds were docked, as an exploratory screen, against three well-characterised and druggable cancer-associated protein targets: the mutant p53 core domain (relevant to the HCT-116 colorectal line), the AKT1 pleckstrin homology domain (relevant to the MDA-MB-231 breast line), and the KRAS switch-II pocket (relevant to the PANC-1 pancreatic line). Because the extract was tested as a mixture, these targets were treated as representative cancer-associated proteins rather than as a strict one-to-one assignment to each cell line. The protein structures used for docking were obtained from the RCSB Protein Data Bank: 6GGB (for mutant p53 Y220C), 1UNQ (for the AKT1 pleckstrin homology domain), and 4LUC (for the KRAS switch-II pocket). The 6GGB structure represents the p53 cancer mutant Y220C in complex with a small-molecule stabiliser, while 1UNQ represents the high-resolution structure of the AKT1/PKBα pleckstrin homology domain bound to Ins [1,3–5]P4.

The rationale for these target choices is as follows. In p53, the Y220C substitution opens a well-defined surface cavity that is absent from the wild-type protein; this cavity is a druggable pocket that has been used to design small-molecule stabilisers of mutant p53 [27], which makes 6GGB a suitable ligand-bound template for docking. HCT-116 cells carry wild-type TP53, and the wild-type core domain does not present a comparable well-defined pocket, so docking against the Y220C cavity was treated as an exploratory model of a ligand-accessible p53 surface rather than as a cell-line-specific mechanism. For AKT1, the PH domain binds phosphatidylinositol-3,4,5-trisphosphate (PIP3) and controls recruitment and activation of the kinase at the plasma membrane; small molecules that occupy this pocket suppress AKT signalling and promote apoptosis [28,29], so 1UNQ offers a defined, ligand-bound site for docking. For KRAS, 4LUC resolves the switch-II allosteric pocket, the druggable site on oncogenic KRAS that was first characterised in the G12C mutant [30] and that is also engaged by the G12D-selective inhibitor MRTX1133 [31]. Since PANC-1 carries the KRAS G12D mutation, a G12D structure such as the MRTX1133-bound complex (PDB 7RPZ) would give the closest match to this cell line, and confirmatory docking against it is a logical next step; the present KRAS analysis is therefore exploratory.

#### Protein preparation.

For each of the selected proteins, the crystal structure was prepared using Protein Preparation Wizard in Schrödinger Maestro. The protein structures were modified by adjusting water molecules, hydrogen atoms, bond orders, and protonation state at the physiological pH. Then, the reformed structures were optimized with the minimum energy level using the Schrödinger force-field workflow.

#### Ligand Preparation.

In order to standardize the geometry of all ligand phytoconstituents before analyzing their potential interactions with receptors, the structures of the three selected compounds were modified as follows: structures were converted to 3D conformations, hydrogen atoms were added, and the possible ionization states under physiological conditions were predicted.

#### Receptor grid generation.

A receptor grid was generated to predict the mechanisms of interaction between ligands and the corresponding binding regions of each protein. For p53 Y220C, the grid was placed around the mutation-associated surface cavity represented in PDB ID 6GGB. For AKT1, the grid was centered around the ligand-binding region of the PH domain in PDB ID 1UNQ. For KRAS, the grid was placed around the switch-II pocket resolved in PDB ID 4LUC. The receptor grid was generated using the Receptor Grid Generation module in Maestro.

#### Molecular docking protocol.

Molecular docking was performed using the Glide module in Schrödinger Maestro. Each ligand was docked independently into each protein grid using standard precision docking. Glide evaluates ligand poses inside the receptor binding site and ranks them using a docking score that approximates the binding favorability of the ligand–protein complex. The docking workflow followed the general Glide protocol, which has been widely used for structure-based virtual screening and ligand–protein binding prediction.

### Statistical analysis

Statistical analysis was performed using Statistical Package for Social Sciences (SPSS) version 22. The dose-response curve was generated using the four-parameter logistic model, a nonlinear regression technique, by modelling the absorbance value as a response against the log value of the dose. The Half-Maximal Inhibitory Concentration (IC_50_) was computed from the dose-response curves, not from the interpolation of a single point. A one-way ANOVA followed by Tukey’s post hoc test was used to compare treatments across multiple cell lines. Statistical significance thresholds were predefined (*p* < 0.05).

## Results

### Plant materials

The plant material was identified by taxonomists at the Royal Society for the Conservation of Nature (RSCN) as *Artemisia -albaherba*.

### MTT-based cell viability and cytotoxicity assay

**Fig 1:**
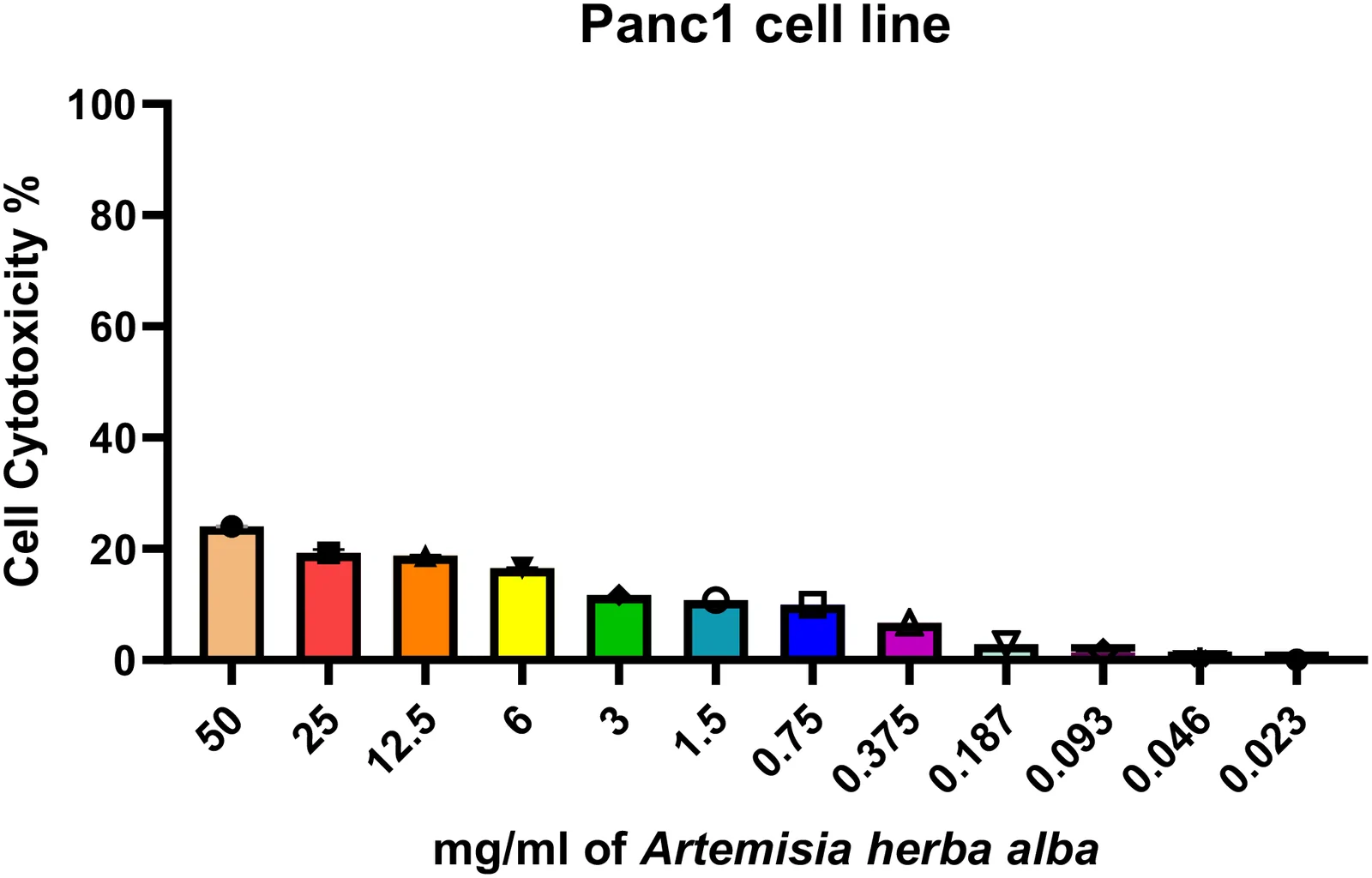
Cytotoxic effects of *A. herb alba* extract on PANC1 cell line. The average of the three measurements ± standard deviation (SD) (n = 3–4 independent repetitions) are used to express the results.

Treating pancreatic cancerous cells with a prepared range of concentrations from the *A. herba-alba* aqueous extract indicated a moderate cytotoxic effect of the crude extract at the tested concentration, and the cells’ viability was inversely related to the treatment concentration. Fig 1 shows the detailed response of the pancreatic cells to the treatment dosages.

**Fig 2:**
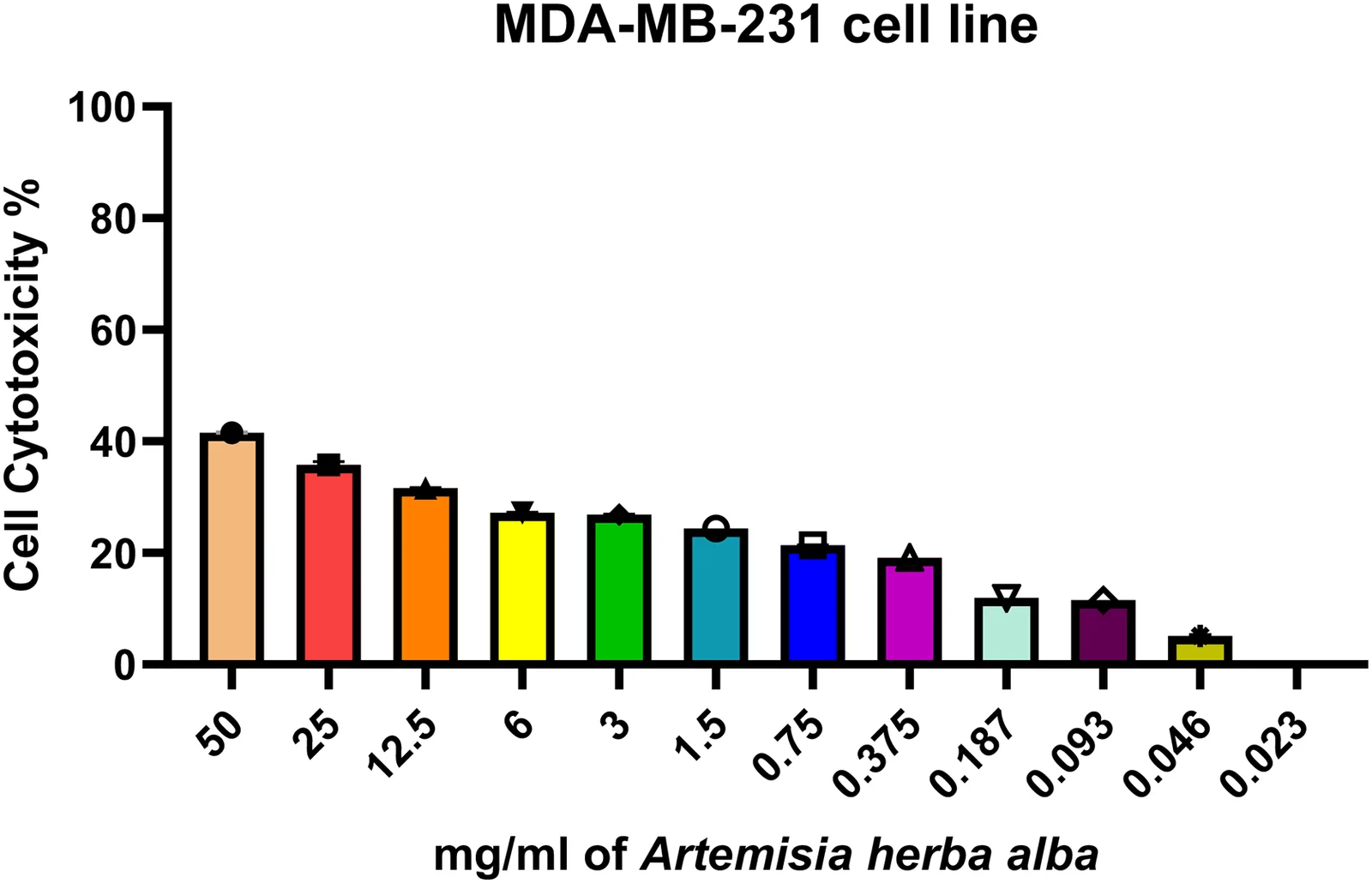
Cytotoxic effects of *A. herb alba* extract on MDA-MB-231 cell line. The average of the three measurements ± standard deviation (SD) (n = 3–4 independent repetitions) are used to express the results.

The Triple-Negative Breast cancer cell line MDA-MB-231 responded to the same range of plant concentrations in a similar pattern. The extract exerted a high level of cytotoxicity against the breast cancerous cells, starting from 50 mg/mL to 0.046 mg/mL doses. Fig 2 shows the exact responses of the breast cancer cells to the tested range of concentrations.

**Fig 3:**
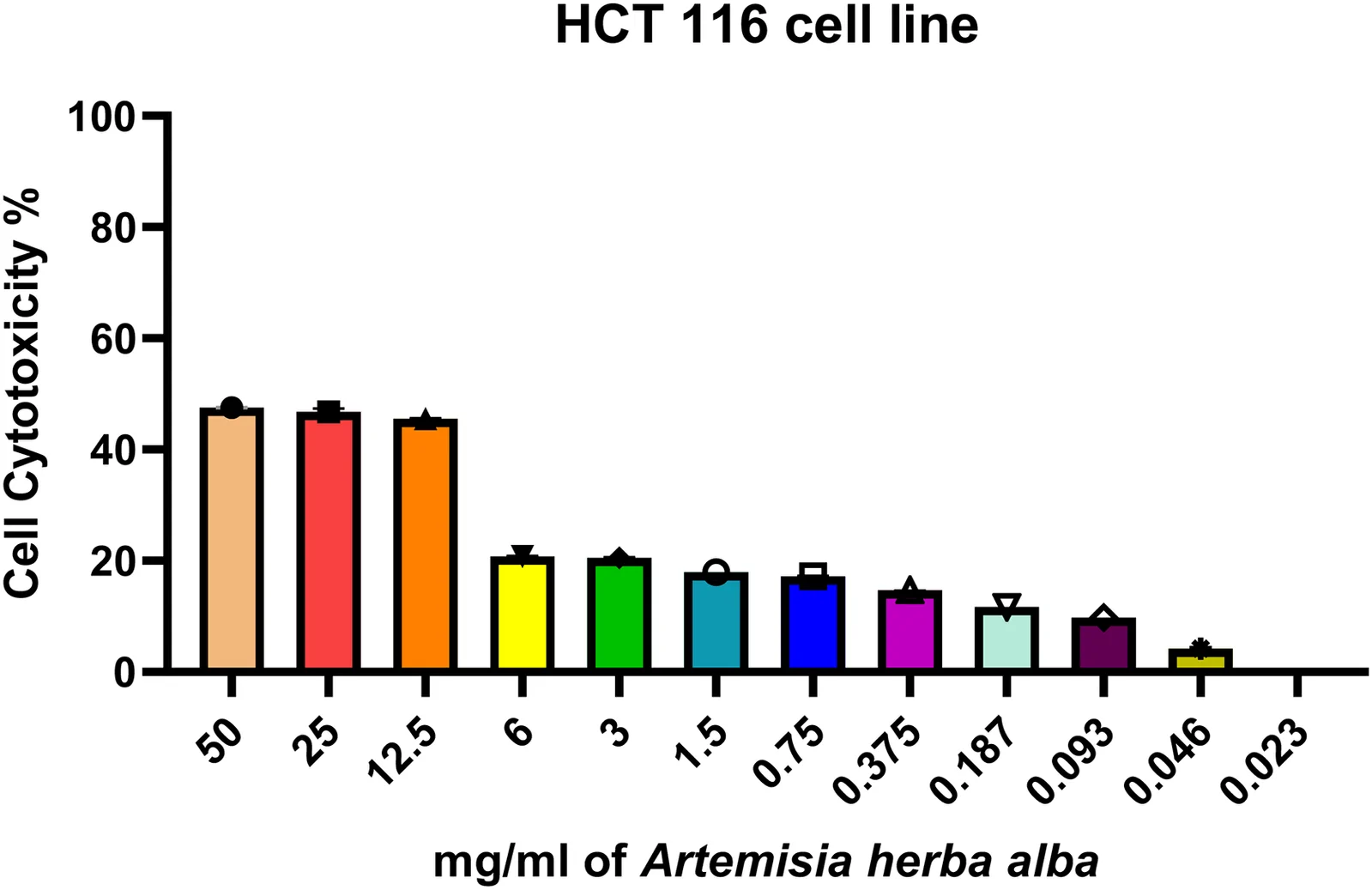
Cytotoxic effects of *Artemisia herb alba* extract on HCT 116 cell line. The average of the three measurements ± standard deviation (SD) (n = 3–4 independent repetitions) are used to express the results.

The viability of Human Colorectal Carcinoma cells HCT 116 was remarkably decreased after treating them with the plant extract in higher doses (12.5–50 mg/mL). Lower concentrations starting from 0.046 to 6 mg/mL plant extract exerted cell cytotoxicity as well, but to a lower degree. The response of HCT to the herbal treatment is illustrated in Fig 3.

**Fig 4:**
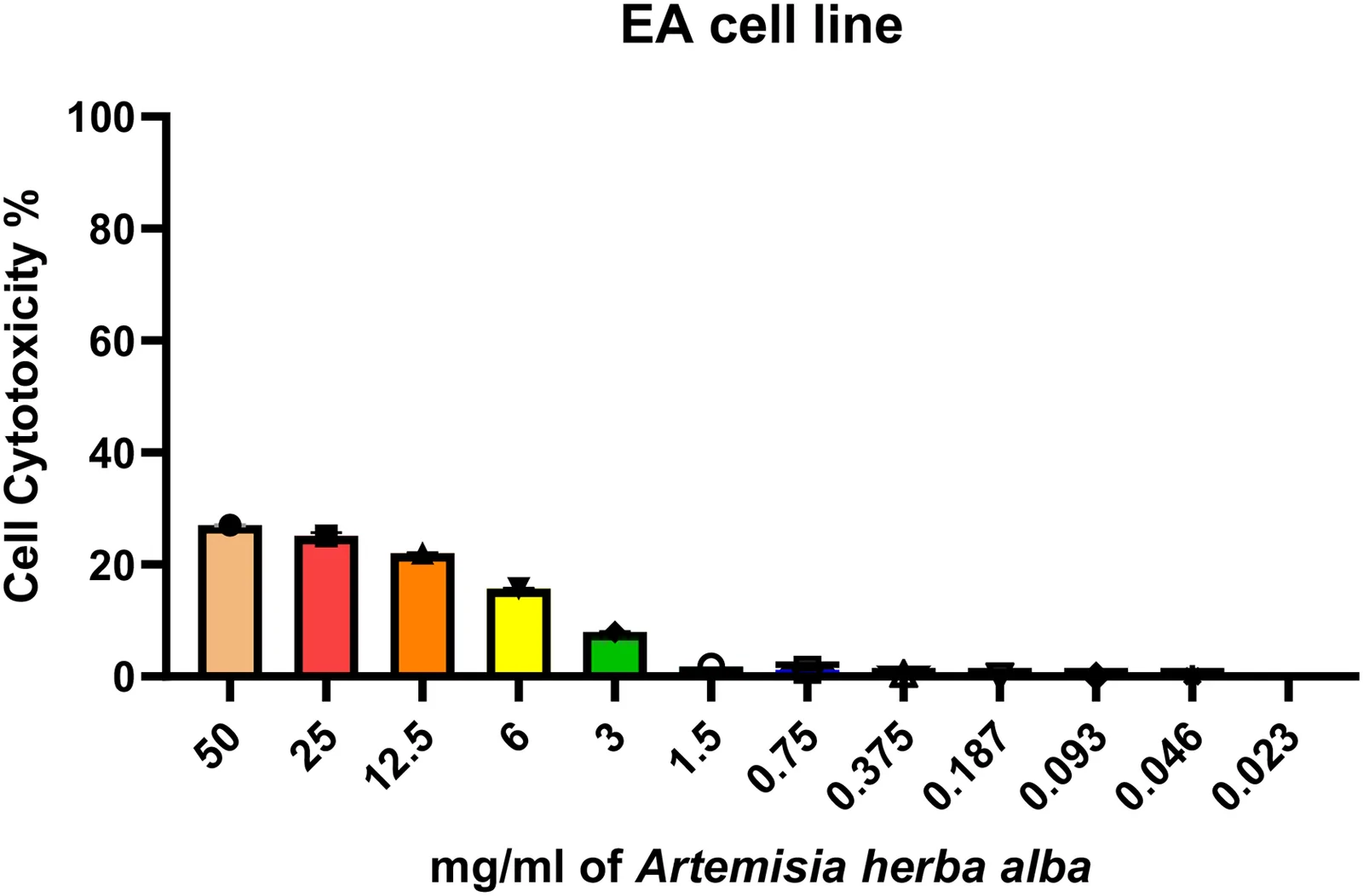
Cytotoxic effects of *Artemisia herb alba* extract on EA normal cell line. The average of the three measurements ± standard deviation (SD) (n = 3–4 independent repetitions) are used to express the results.

Contrariwise, the herbal extract didn’t show a remarkable cytotoxicity at the used range of concentrations against the normal Human Umbilical Vein Endothelial Cell line (HUVEC). The cell survival after the treatment indicated a low level of toxicity to normal cells. The higher tested concentrations (12–50 mg/mL) have around 20–23% of cells’ cytotoxicity, and no cytotoxic effect is observed at 1.5 mg/mL, indicating its biocompatibility. Fig 4 illustrates the response of EA cells to the prepared herbal remedy.

**Fig 5:**
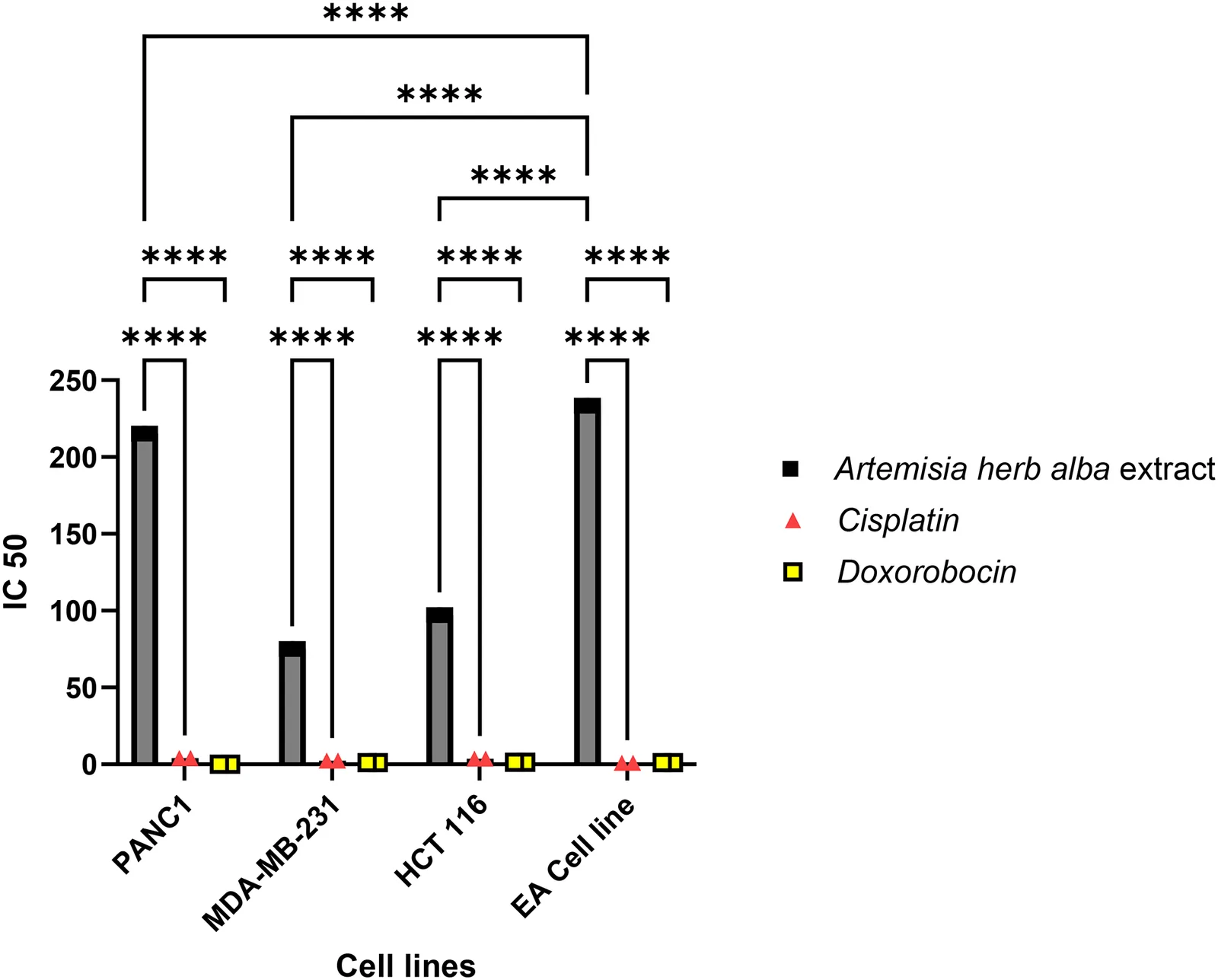
IC₅₀ values of *A. herb alba* extract compared with standard /chemotherapeutic drugs in different cell lines. Bar graph showing the IC₅₀ (µg/mL) of *A. herb alba* extract (black bars) in PANC-1, MDA-MB-231, HCT-116 and EA cell line, in comparison with cisplatin (red triangles) and doxorubicin (yellow squares). **** indicates statistically significant differences at *P* < 0.0001 between the indicated groups.

To enhance the robustness of the used method and the interpretation of the obtained results, a comparative Figure was added to present the IC_50_ values of the plant extract alongside those of the two conventional anticancer drugs in treated cancerous and normal cells (Fig 5). The MTT assay was performed at the same experimental conditions using the conventional chemotherapeutic agents, cisplatin and doxorubicin, as positive controls. The graph shows notably higher IC_50_ values for the extract against the selected cell lines than for the anticancer drugs. This indicates a significantly lower cytotoxicity potential of the Artemisia crude extract compared to the tested chemotherapeutic agents.

The computed IC_50_ values for the extract in PANC1 and EA cells are approximately 220–240 µg/mL, and in MDA-MB-231 and HCT-116 cells are 80–100 µg/mL. On the other hand, the IC_50_ values for both cisplatin and doxorubicin in the selected cells range from 1 to 5 µg/mL. Statistical analysis revealed highly significant differences (*P* < 0.0001) between the extract and each of the reference drugs in all cell lines.

### UHPLC/MS-MS analysis

**Fig 6:**
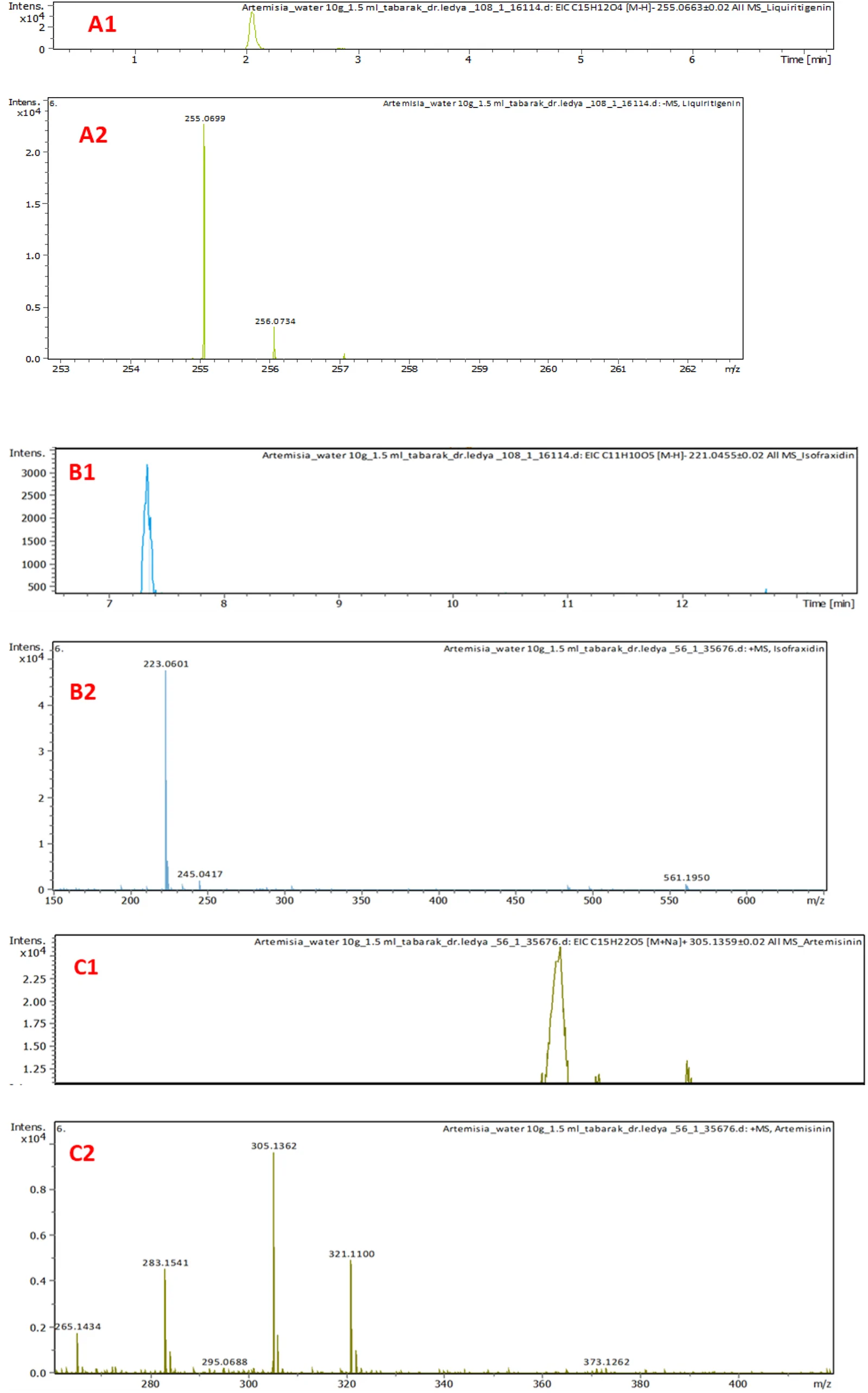
Ion chromatogram and mass spectra for Liquiritigenin (A), isofraxedin (B), and artemisinin (C).

The compounds of interest were detected and identified in the aqueous extract based on their retention time and mass to charge ratio (m/z), as shown in Table 1 and Fig 6. The detailed LC/MS analysis is shown in supplementary I.

**Table 1:** The LC/MS data of artemisinin, isofraxidin, and liquiritigenin in *A. herba* alba extract.

Rt [min]	m/z meas.	Theoretical mass	Ions	Name	Molecular Formula	Intensity
4.72	305.1359	282.1467	[M+Na]^+,^ [M+H]^+^, [M+H-H2O]^+^, [M+K]^+^	Artemisinin	C_15_H_22_O_5_	25610
6.35	223.0602	222.0529	[M+H]^+^	Isofraxidin	C_11_H_10_O_5_	84878
2.05	255.0874	256.0947	[M-H]^-^	Liquiritigenin	C_15_H_22_O_4_	34046

### Molecular docking analysis

Molecular docking was performed to evaluate the predicted binding of three major *Artemisia -albaherba* phytochemicals, namely artemisinin, isofraxidin, and liquiritigenin, against three cancer-associated targets representing the selected cell-line models: AKT1 for the MDA cell line, the KRAS switch-II pocket for the PANC-1 cell line, and the TP53/p53 mutant structure for the HCT-116 cell line. The docking was performed using Glide standard precision docking, where more negative Glide docking scores indicate more favorable predicted ligand–protein binding; however, these scores should be interpreted as relative docking estimates rather than experimentally measured binding free energies. Glide is designed to sample ligand conformational, orientational, and positional space within the binding site and rank predicted poses using GlideScore-based scoring functions.

The strongest predicted interaction overall was observed for the TP53 system, where isofraxidin and liquiritigenin showed markedly favorable docking scores of −7.975 and −7.951 kcal/mol, respectively. In contrast, artemisinin showed weaker predicted affinity across the three targets, with docking scores ranging from −3.223 to −3.663 kcal/mol. Among the three protein targets, the AKT1 structure used was the pleckstrin homology domain of protein kinase B/AKT bound to Ins(1,3,4,5)P4, while the TP53 structure corresponded to the p53 Y220C mutant complexed with a small-molecule stabilizer. Table 2 illustrates the docking scores and the relative rank between the selected phytoconstituents and the corresponding target proteins in the cancerous cells.

**Table 2:** The docking score and the relative rank of selected phytoconstituents and theirs corresponding target proteins in the cancerous cells.

Target protein	PDB Id	Compound	Docking score, kcal/mol	Relative rank within target
**TP53/p53 mutant**	6GGB	Isofraxidin	−7.975	1
**TP53/p53 mutant**	6GGB	Liquiritigenin	−7.951	2
**TP53/p53 mutant**	6GGB	Artemisinin	−3.223	3
**AKT1**	1UNQ	Isofraxidin	−5.729	1
**AKT1**	1UNQ	Liquiritigenin	−3.952	2
**AKT1**	1UNQ	Artemisinin	−3.663	3
**KRAS G12D**	4LUC	Liquiritigenin	−5.936	1
**KRAS G12D**	4LUC	Isofraxidin	−4.270	2
**KRAS G12D**	4LUC	Artemisinin	−3.453	3

The simulated interaction with the AKT1 receptor:

AKT1-Isofraxidin: the computed docking score (−5.729 Kcal/mol) indicated the strongest binding into the target compared to the other tested compounds. The interaction was found with the residues LYS14, GLU17, TYR18, ILE19, ARG23, ARG25, LEU52, ASN53, and ARG86. Water-mediated interactions through several polar functional groups, including carbonyl, hydroxyl, and methoxyl of isofraxidin. Moreover, aromatic/hydrophobic interactions within the pocket support the polar binding and further strengthen binding potential.

AKT1-Artemisinin: the computed docking score (−3.663 Kcal/mol) indicated weaker binding of artemisinin into AKT1 compared to the other compounds. The position of artemisinin was shown by the 2D map near the residues PRO51, LEU52, ASN53, ASN54, and GLN79. A water-mediated interaction was seen through the lactone or carbonyl oxygen of artemisinin. The nearby hydrophobic residues, especially PRO51 and LEU52, seemed to help fill the pocket. Still, the interaction pattern was limited and showed few direct polar contacts, which matches its lower docking score.

AKT1-Liquiritigenin: the computed docking score (−3.952 Kcal/mol) indicated that its binding affinity is weaker than that of isofraxidin and stronger than artimisinin. The compound positioned itself in the polar and aromatic regions relating to LYS14, GLU17, GLY16, TYR18, ILE19, ARG23, ASN53, PHE55, and ARG86. The polar interactions involve the phenolic and carbonyl functional groups, include interactions with ARG23, LYS14, and ARG86, while the hydrophobic/aromatic residues include interactions with TYR18, ILE19, and PHE55.

The simulated interaction with the KRAS switch-II pocket:

KRAS-liquiritigenin: the computed docking score (−5.936 Kcal/mol) indicated the strongest binding affinity to KRAS compared to the other tested compounds. The residue environment includes GLY10, ALA11, CYS12, PRO34, ALA59, GLY60, GLN61, GLU62, GLU63, ARG68, TYR96, and GLN99. The polar interaction involves phenolic, hydroxyl, and heterocyclic oxygen functional groups. The interaction with CYS12, GLN61, ARG68, and GLN99 explains the strong and stable interaction within the receptor pocket.

KRAS-isofraxidin: the computed docking score (−4.270 Kcal/mol) indicated stronger binding affinity than artimisinin and weaker than liquiritigenin. The ligand positioned with residues including THR35, ILE36, GLU37, ASP38, GLY60, GLN61, MET67, and TYR71. The polar interaction involves the carbonyl and hydroxyl functional groups of the ligand with GLN61 and THR35. The aromatic coumarin system was positioned within a mixed polar–hydrophobic environment, resulting in a more stable binding pose than that of artemisinin.

KRAS-artemisinin: the computed docking score (−3.453 Kcal/mol) indicated moderate binding affinity that is relatively weaker than the other two tested compounds. Its positioned around the residues ARG68, TYR64, GLU63, HIE95, GLU98, GLN99, and ARG102. The generated map showed a limited interaction with weak polar contacts and complementing the shape.

The simulated interaction with the TP53/p53 mutant:

TP53/p53- isofraxidin: the computed docking score (−7.975 Kcal/mol) indicated strong binding affinity, which is relatively comparable to liquiritigenin, and much stronger than that of artimisinin. The interaction occurs around a TP53 pocket similarily to that of liquiritigenin. The polar interaction is between the phenolic hydroxyl and carbonyl groups with LEU145 and CYS220 residues, while the non-polar interactions between the aromatic residues stabilize the aromatic scaffold.

TP53/p53-liquiritigenin: the computed docking score (−7.951 Kcal/mol) indicated strong binding affinity, which is relatively comparable to that of isofraxidin and much stronger than that of artimisinin. The interaction occurs around a TP53 pocket surrounded by the residues PHE109, LEU145, TRP146, VAL147, THR150, PRO151, PRO153, THR155, CYS220, GLU221, PRO222, PRO223, ASP228, CYS229, and THR230. The generated 2D map predicted a hydrophobic/aromatic interaction with LEU145, TRP146, VAL147, PRO151, and l PRO153, while polar residues such as THR150, THR155, CYS220, GLU221, ASP228, and THR230 surrounded the ligand and may contribute to additional stabilization.

**Fig 7:**
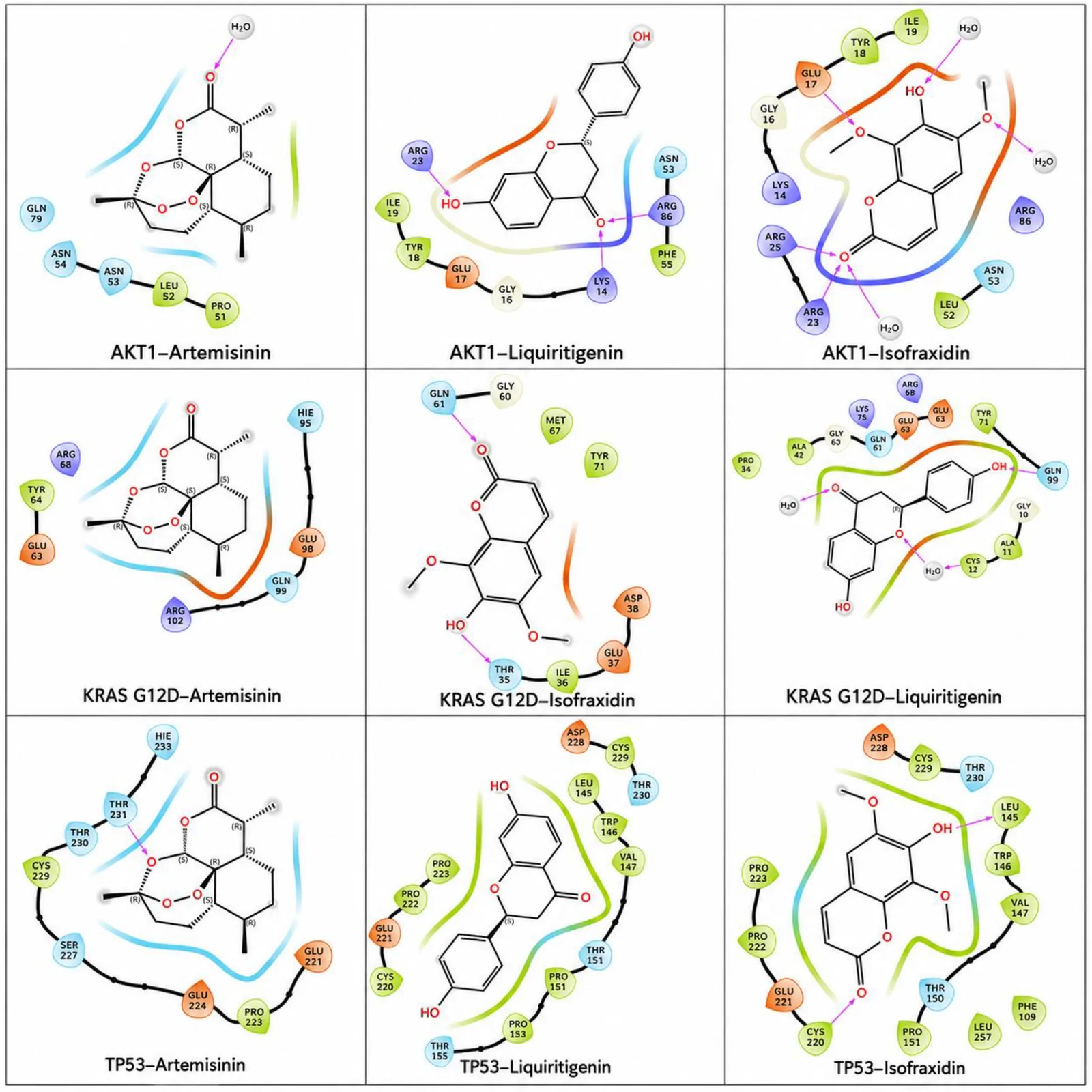
Shows the 2D-interaction mas between the three tested ligands isofraxidin, liquiritigenin, and artemisinin against three cancer-associated protein targets corresponding to the three investigated cancer cell lines.

TP53/p53-artemisinin: the computed docking score (−3.223 Kcal/mol) indicated weaker binding affinity than the other tested ligands. The interaction points were found to be limited to polar binding with the THR231 residue, and conformational accommodation within the pocket. Fig 7 shows the 2D-interaction mas between the three tested ligands isofraxidin, liquiritigenin, and artemisinin against three cancer-associated protein targets corresponding to the three investigated cancer cell lines.

To sum up, the docking analysis predicted that liquiritigenin and isofraxidin bind the selected cancer-associated targets more favourably than artemisinin. Because the extract, and not the isolated compounds, was tested in the cell assays, these two phenolic compounds are best regarded as the most promising candidates for follow-up work rather than as confirmed effectors.

## Discussion

In spite of the extensive studies on the biological activities of *A. herba-alba* extracts and the investigation of its active components, limited research was done to explore the antitumor activity of its aqueous extract and the safety profile of the herbal tea. The current research is concerned with studying the effect of the *Artemisia* plant, which grows widely in South Jordan, on normal and cancerous cell lines to justify the current folk use and to investigate the potential of synthesizing an effective and safe anticancer agent based on biologically active components in the plants’ aerial parts.

As demonstrated in previously published articles, aerial parts of *A. herba-alba* grown in different endemic regions contain several groups of active metabolites, including polyphenols, volatile oils, and terpenes. The volatile oils consist mainly of 1,8-cineole, β-thujone, camphor, and chrysanthenone [32]. In addition to the well-known components santonin and artemisinin [33].

Santonin and artemisinin are naturally occurring sesquiterpene lactones with antimalarial activity. The pure compounds showed potent antitumor activity against several cell lines after *in vitro* studies [34]. Several mechanisms were predicted to explain its cytotoxicity, including a free radical generator to break the endoperoxide bond in cancerous cells, apoptosis induced by free radicals, damage of the genetic material deoxyribonucleic acid (DNA), modulating the responding abilities of the receptors, inhibiting angiogenesis and metastasis [35].

Likewise, Artemisia oil was investigated for its antitumor activity. The bioactive compounds of *A. campestris* essential oil, linalyl acetate, geranyl acetate, and eucalyptol exhibit various biological activities, including anti-oxidation, anti-inflammation, and potential antitumor activities. The essential oil extracted from Algerian habitat was tested for potential anticancer activity, and docking analysis revealed a strong binding affinity of the phytoconstituent 3-cyclopentyl-*N*-(2-(3,4-dimethoxyphenyl)ethyl) to phosphoinositide 3-kinase gamma. The enzyme represents a target for pancreatic cancer treatment [31]. This emphasises the hypothesis that the phytoconstituents of Artemisia possess cytotoxic properties.

The obtained results are consistent with previous findings; It is assumed that the measured activity might have been partly due to sesquiterpene lactones, phenolic compounds and others that we detected in the extracts. Nevertheless, further research is needed to determine whether there is a definitive relationship between the compounds in the extract and biological activity. The aqueous extract displayed modest but significant cytotoxicity against the evaluated cell lines with an enhanced activity on the cell lines HCT-116 and MDA-MB-231. This activity although low compared with those generated by chemotherapeutic agents, it appeared to be selective towards tumor cells compared to the HUVEC cells. These results are preliminary and further studies will be needed to evaluate the actual impact of these purified fractions and the mechanism by which these effect occur.

The effect on pancreatic cancer cells is less potent, as the highest tested dose shows around 20% cytotoxicity, with a gradual decline in cytotoxicity at lower concentrations.

Previous results support the current assumption that Artemisia might incorporated as a potential anticancer remedy. For example, the ethyl-acetate extract of *A. campestris has been tested against* anti-leukemic activities [36]. Moreover, methanol and ethyl acetate extracts of Tunisian *A. campestris* showed significant impact on metastatic breast cancer and multiple myeloma after in vitro studies. These effects were attributed to the polyphenolic content of the extract [37].

The concentration range employed in the present study is admittedly relatively high when considering the use of the purified natural product. Nevertheless, it must be considered that the material utilized in this study was a crude aqueous extract which contained a varied compound mixture where some compounds might have existed at relatively low levels, and hence, much higher concentrations would have been needed to assess some potential biological effect. The range is also compatible to that observed in several reports on crude plant extracts.

The interpretation of the obtained results parallels previous reports on the antitumor activity of other Artemisia species or of the same species cultivated in other regions. *A. absinthium* inhibits the proliferation of the MDA-MB-231 breast cancer cell line by regulating Bcl-2 family proteins and MEK/MAPK signaling, thereby inducing apoptosis [38]. Coincidentally, a dose-dependent effect of the *A. absinthium* extract on HS578T breast cancer cells was reported [39].

The observed effect of *A. herbal alba* on colon cancer cells is consistent with previously published findings on the cytotoxic effects of other Artemisia species, namely *A. vulgaris* and *A. alba Turra*. Non-polar extract of A. vulgaris showed a weaker anti-colon cancer effect compared to *A. alba turra* extract [40]. A potential antimetastatic effect of bioactive compounds isolated from the ethanolic extract of *A. annua* exhibited a dose- and time-dependent pattern as reported by Zhou’s research group [41]. This effect is synchronized with the current findings of the tested extract against the pancreatic cancerous cell line.

Nevertheless, the measured cytotoxicity of natural products as crude extracts couldn’t be comparable to conventional chemotherapeutic agents. But phytomedicines still have a promising potential as a sustainable and safer alternative. Placing the cytotoxicity of Artemisia aqueous extract in direct context with standard anticancer drugs, cisplatin and doxorubicin, strengthens confidence in the biological outcomes, even though it is highly expected that their cytotoxic effects will not be comparable. The statistical analysis of the results indicated measurable cytotoxicity of the natural remedy, despite its activity being considerably inferior to that of the chemotherapeutic references.

To further support the obtained results and to predict the mechanism of the extract’s cytotoxicity, three active phytoconstituents that have been identified in the extract in remarkable amounts were docked against the protein targets of each selected cell line, and the binding affinity and pattern were examined. Isofraxidin, a coumarin, was abundant in the examined aqueous extract. It has been detected in other artemisia species such as *A. capillaris*. The isolated compound showed hyperpigmentation activity by regulating the melanin synthesis cascade and increasing the expression of tyrosinase and other melanogenesis factors [42]. Results from previously published research revealed that isofraxidin inhibits cell apoptosis and regulates the expression of lung cancer-associated proteins [43]. Isofraxidin was found to be a promising agent for treating pathological osteogenesis in the autoimmune disorder ankylosing spondylitis by inhibiting osteoblast viability and altering molecular pathways [44]. Accordingly, Isofraxin is a biologically active compound and might contribute to the cytotoxicity effect of *A. herba alba*. This is consistent with the docking analysis, which predicted a strong binding affinity to the protein targets associated with the selected cell lines, through both polar and non-polar interactions.

It was interesting to detect liquiritigenin (7,4’-Dihydroxyflavanone) in the artemisia extract, even at a low-intensity peak. liquiritigenin it is mainly a component of *Glycyrrhiza spp* [45].. A closely related flavone, (5,4-dihydroxyflavone) was detected and identified in the ethyl acetate extract of *A. vulgaris* mugwort [46].

Liquiritigenin has demonstrated anticancer mechanisms across various cancer types. It exerts an inhibitory effect on bladder cancer proliferation and migration, and induces autophagy-related apoptosis through the PI3K/AKT/mTOR pathway in oral cancer [47,48]. The obtained results align with previous findings and predict an anticancer effect against pancreatic, colorectal, and breast cancer through both water-mediated and non-polar interactions with the TP53, AKT1, and KRAS target proteins. These docking results are predictive and hypothesis-generating: because the cellular assays used the whole extract rather than the purified compounds, the individual contribution of isofraxidin and liquiritigenin to the observed cytotoxicity still needs to be confirmed by testing the isolated compounds and by direct target-engagement experiments [49].

The phenolic constituents Isofraxidin and liquiritigenin may contribute to the cytotoxicity of the crude artemisia extract. Specifically, this relationship is evident in their ability to form strong interactions with the binding pockets of the selected target proteins either via hydrophobic interactions with their aromatic scaffolds or via hydrophilic interactions involving their multiple hydrogen-bond acceptor/donor groups. In contrast, artemisinin, despite its characteristic peroxide-containing sesquiterpene lactone structure, showed weaker docking scores and fewer stabilizing contacts across the three tested target proteins.

One shortcoming of this study, the maximal inhibition found within the tested dose range did not exceed 50% in one cell line; hence the computed IC_50_ should be interpreted as an estimated based on modeling rather than achieved inhibition concentrations at experimentally obtained inhibitory doses; additionally, the biological testing was solely confined to the MTT assay, which assesses metabolic viability and not cell death, thus this indirect cytotoxicity finding did not show any indications of any apoptosis, necrosis, cell cycle arrest, or senescence mechanism of growth arrest. So, the mechanism of the observed effect is yet unknown and to investigate further, studies using Annexin V/PI apoptosis staining, measuring caspase activation, cell cycle progression, reactive oxygen species (ROS) measurement, invasion assays and migration assays could be beneficial in defining the precise mechanism of the tested extract.

## Conclusion

Medicinal plants have been extensively studied for their biological properties and potential therapeutic activities. Cancer conventional medicines faced challenges due to resistance development, adverse effects, increased cost, and poor selectivity. The current study showed a preliminary finding regarding the selective cytotoxic effect of *A. herba alba* aqueous crude extract against the multiple cancerous cell lines namely: Triple Negative Breast cancer cell line, Human Pancreatic Ductal Adenocarcinoma cell line, and Human Colorectal Carcinoma cell line. The herbal treatment showed moderate cytotoxic activity towards colon and breast cancer cell lines, nevertheless, it showed weaker activity on the pancreatic cells. The low toxicity on the normal epithelial cell lines indicates biocompatibility and the safety of the herbal extract. The docking simulation studies suggest that the phenolic compounds isofraxidin and liquiritigenin may contribute to the cytotoxicity of the extract, since they showed favourable predicted binding to the selected target proteins, namely TP53, AKT1, and KRAS. As the compounds were not tested individually, this contribution remains a hypothesis to be confirmed experimentally. The interaction with the binding pocket of each target protein was shown to involve a water-mediated polar interaction and hydrophobic attraction via the aromatic scaffold. Further research to predict the exact anticancer mechanism of action and to thoroughly examine the safety profile is encouraged.

## Supporting information

S1 File HPLC/MS analysis results (XLSX)
